# Colonic mucosal and serum expression of microRNAs in canine large intestinal inflammatory bowel disease

**DOI:** 10.1186/s12917-020-02287-6

**Published:** 2020-02-22

**Authors:** Alexandros Ο. Konstantinidis, Dimitra Pardali, Katerina K. Adamama-Moraitou, Maria Gazouli, Chrysostomos I. Dovas, Evangelia Legaki, Georgia D. Brellou, Ioannis Savvas, Albert E. Jergens, Timoleon S. Rallis, Karin Allenspach

**Affiliations:** 1 Companion Animal Clinic (Medicine Unit), School of Veterinary Medicine, Faculty of Health Sciences, Aristotle University of Thessaloniki, Thessaloniki, Greece; 2 Diagnostic Laboratory, School of Veterinary Medicine, Faculty of Health Sciences, Aristotle University of Thessaloniki, Thessaloniki, Greece; 3 Laboratory of Biology, School of Medicine, National and Kapodistrian University of Athens, Athens, Greece; 4 Laboratory of Pathology, School of Veterinary Medicine, Faculty of Health Sciences, Aristotle University of Thessaloniki, Thessaloniki, Greece; 5 Companion Animal Clinic (Anesthesia and Intensive Care Unit), School of Veterinary Medicine, Faculty of Health Sciences, Aristotle University of Thessaloniki, Thessaloniki, Greece; 6 Departments of Veterinary Clinical Sciences, Iowa State University College of Veterinary Medicine, Ames, IA USA

**Keywords:** Biomarker, Dog, Inflammatory bowel disease, microRNAs

## Abstract

**Background:**

Canine inflammatory bowel disease (IBD) is a group of chronic gastrointestinal (GI) disorders of still largely unknown etiology. Canine IBD diagnosis is time-consuming and costly as other diseases with similar signs should be initially excluded. In human IBD microRNA (miR) expression changes have been reported in GI mucosa and blood. Thus, there is a possibility that miRs may provide insight into disease pathogenesis, diagnosis and even treatment of canine IBD. The aim of this study was to determine the colonic mucosal and serum relative expression of a miRs panel in dogs with large intestinal IBD and healthy control dogs.

**Results:**

Compared to healthy control dogs, dogs with large intestinal IBD showed significantly increased relative expression of miR-16, miR-21, miR-122 and miR-147 in the colonic mucosa and serum, while the relative expression of miR-185, miR-192 and miR-223 was significantly decreased. Relative expression of miR-146a was significantly increased only in the serum of dogs with large intestinal IBD. Furthermore, serum miR-192 and miR-223 relative expression correlated to disease activity and endoscopic score, respectively.

**Conclusion:**

Our data suggest the existence of dysregulated miRs expression patterns in canine IBD and support the potential future use of serum miRs as useful noninvasive biomarkers.

## Background

Canine inflammatory bowel disease (IBD) is a group of chronic or recurrent gastrointestinal (GI) disorders with histopathologic evidence of inflammation in intestinal and/or colonic tissue. The exact pathogenesis of these disorders is still unknown; however, genetic factors, intestinal microbiota, environmental factors and deregulated host immune response may contribute to the pathogenesis of the disease [1]. The prevalence of IBD is unknown, but it is the most common histopathologic diagnosis of dogs with chronic vomiting and/or diarrhea. The term IBD should be used if no underlying cause for the inflammation can be documented [2]. Histopathologic confirmation of intestinal inflammation is the gold standard of diagnosis. The establishment of non-invasive biomarkers would help diagnosis, prognosis and disease-monitoring.

According to Bartel (2009), microRNAs (miRs) are small (≈22 nucleotides in length), non-coding RNA molecules with post-transcriptional gene regulatory function via translational repression or messenger (m)RNA degradation [3]. miRs are highly conserved even among distantly related species and are involved in the regulation of many biological processes such as the cell life cycle [4–6]. miRs have a complementary binding site on numerous mRNAs and each gene can be regulated by many miRs [3, 4, 7]. In humans, many studies indicate several miRs as regulators of important pathways of the immune response and immune cell development, which are crucial to the pathogenesis of a variety of diseases, including IBD [8, 9]. In addition, miRs are released extracellularly by immune and non-immune cells. Circulating miRs were firstly identified in serum and plasma samples and later in other body fluids (saliva, urine and semen) [10–14]. miRs are remarkably stable in rough conditions (low or high pH, extended storage conditions, boiling, and several freeze-thaw cycles) and are early dysregulated during the course of a broad spectrum of diseases ranging from neoplastic to inflammatory [10, 11, 15–19]. Their expression levels can be easily measured by quantitative polymerase chain reaction (PCR), which has high-precision signal amplification, allowing measurement of very low amounts of miRs in small volumes of body fluids. Furthermore, the expression of some miRs is specific to tissues, and type and/or stage of the disease [20–25].

At present, in small animal medicine, little is known about miRs expression in health and disease, including canine IBD. Regarding other canine digestive system diseases some studies have evaluated miRs expression, especially miR-122, and its dysregulation in hepatobiliary diseases [26, 27]. In human IBD, more than 100 miRs have shown altered expression in GI mucosa and/or peripheral blood and serum (circulating miRs) [28–31], with the latter being a promising non-invasive biomarker for the disease. The aim of the present study was to determine the colonic mucosal and serum relative expression of a selected panel of miRs (miR-16, miR-21, miR-122, miR-146a, miR-147, miR-185, miR-192 and miR-223), as well as to evaluate their correlation in dogs with the degree of histopathological inflammation compared to healthy controls. The miRs selection for this study was based on a combination of their role in the intestinal inflammation in humans and the relevant findings of studies on canine IBD pathogenesis regarding changes in the mucosal immune system [1, 8, 25, 28, 32–36]. Possible correlations of clinical disease activity, endoscopic grading and histopathologic scoring with miRs relative expression in colonic mucosa and serum in dogs with large intestinal IBD were also investigated.

## Results

### Characteristics of dogs with large intestinal IBD and healthy control dogs

Epidemiological data, canine chronic enteropathy clinical activity index (CCECAI) score and quantitative colonoscopy score of 26 dogs with large intestinal IBD of the study are presented in Table 1. Median age of the dogs was 7 years (range = 1–15 years). Most of the dogs (7/26) with large intestinal IBD were mongrels. Body weight ranged from 1.72 to 36.5 kg (median = 23 kg). Median CCECAI score of dogs with IBD was 7 (range = 4–11). All dogs with large intestinal IBD included in this study had clinical signs of large bowel diarrhea, with mucus and fresh blood in the feces as well as tenesmus. Most dogs with large intestinal IBD had moderate CCECAI score (10/26, range = 6–8), 9/26 had mild (range = 4–5) and 7/26 had severe (range = 9–11). Median quantitative endoscopy score of dogs with IBD was 4 (range = 1–6). All dogs with large intestinal IBD had evidence of primarily lymphoplasmacytic inflammation (Table 2).

**Table 1:** Epidemiological data, canine chronic enteropathy clinical activity index (CCECAI) score and quantitative endoscopy score of 26 dogs with large intestinal inflammatory bowel disease (IBD)

Case No.	Breed	Sex	Body weight (kg)	Age (years)	CCECAI score	Quantitative colonoscopy score
IBD1	Mongrel	M	7.6	2.5	7	3
IBD2	Chihuahua	M	1.72	3	11	5
IBD3	Pitbull	F	24	1.2	5	3
IBD4	Chihuahua	Fs	2.5	6	8	6
IBD5	Mini Pinscher	M	7.5	9.5	8	4
IBD6	Mongrel	M	8	5.5	7	4
IBD7	Mongrel	Fs	25	12	5	2
IBD8	Mini Pinscher	M	1.85	7	10	4
IBD9	Labrador retriever	Fs	21.2	1.5	9	4
IBD10	WHWT	M	8	3	5	6
IBD11	Mongrel	Fs	25.3	6	7	4
IBD12	Mongrel	M	27.9	4	6	5
IBD13	Mongrel	F	7.15	2.5	7	2
IBD14	Maltese	M	1.95	11	9	4
IBD15	Pitbull	M	25.5	13	4	4
IBD16	Mongrel	M	9.5	8	4	2
IBD17	Pug	Fs	7.95	12.5	7	4
IBD18	Pitbull	M	26.8	3.5	6	1
IBD19	WHWT	M	8.5	11	4	2
IBD20	Mongrel	Fs	23	7	5	3
IBD21	Yorkshire Terrier	M	3.4	8.5	7	4
IBD22	Maltese	Fs	4.25	7	10	3
IBD23	GSD	Mn	36.5	1.5	5	3
IBD24	Mongrel	Fs	22.1	6	4	1
IBD25	Yorkshire Terrier	Fs	4.1	15	11	5
IBD26	Bichon Frise	Fs	7.1	12	10	2

*M* male, *Mn* male neutered, *F* female, *Fs* female spayed, *WHWT* West Highland White Terrier, *GSD* German Shepherd dog

**Table 2:** Histopathologic scores of 26 dogs with large intestinal inflammatory bowel disease (IBD)

Case	Surface epithelial injury	Crypt hyperplasia	Crypt dilation and distortion	Fibrosis and atrophy	Lamina propria lymphocytes/plasma cells	Lamina propria neutrophils	Lamina propria eosinophils	Lamina propria macrophages
IBD1	1	3	0	2	3	1	0	0
IBD2	1	2	0	2	2	0	0	1
IBD3	1	1	1	1	1	0	0	0
IBD4	0	2	1	1	1	0	1	1
IBD5	0	2	2	2	1	0	0	1
IBD6	1	1	0	1	2	1	0	1
IBD7	1	1	0	1	2	0	0	0
IBD8	2	3	3	2	3	1	0	1
IBD9	1	1	1	1	1	1	0	1
IBD10	2	2	2	2	2	1	0	1
IBD11	0	2	2	0	1	1	0	0
IBD12	1	2	1	1	2	0	0	1
IBD13	2	2	1	2	2	1	0	1
IBD14	1	3	2	2	2	0	0	0
IBD15	0	2	1	1	2	0	0	0
IBD16	1	2	2	0	1	0	0	0
IBD17	1	2	1	0	1	0	0	1
IBD18	1	2	2	1	1	0	0	1
IBD19	1	2	2	1	2	0	0	1
IBD20	1	1	1	0	2	0	0	0
IBD21	1	2	2	1	1	0	0	0
IBD22	1	2	1	1	2	0	1	1
IBD23	1	2	2	1	2	0	0	0
IBD24	1	2	2	1	1	0	0	1
IBD25	0	1	2	3	2	1	0	1
IBD26	1	2	0	2	2	1	0	0

Table 3 shows the epidemiological data of controls. Their median age was 3 years (range = 2–7 years). Most of them (9/16) were mongrels. Body weight ranged from 5 to 40 kg (median = 27.5 kg).

**Table 3:** Epidemiological data of 16 healthy dogs (C)

Control No.	Breed	Sex	Age (years)	Body weight (kg)
C1	Golden Retriever	M	7	35
C2	Mongrel	M	2	32
C3	Mongrel	M	2	26
C4	Mongrel	M	2	29
C5	Mongrel	Fs	2	24
C6	Boxer	M	3	21
C7	Pekingese	Fs	7	8
C8	Mongrel	Fs	5	26
C9	Mongrel	Fs	3	28
C10	GSD	Fs	4	27
C11	Greek Shepherd	M	2	40
C12	Mongrel	Fs	7	5
C13	Belgian Shepherd	F	7	20
C14	Labrador	Fs	4	30
C15	Mongrel	M	2.5	28
C16	Mongrel	M	2.5	31

*M* male, *F* female, *Fs* female spayed, *GSD* German Shepherd dog

### Relative expression of microRNAs in the colonic mucosa and serum of dogs with large intestinal IBD and healthy dogs

In all dogs with large intestinal IBD, colonic mucosal miRs expression analysis was assessed, while serum miRs expression analysis was available for a majority of the dogs with large intestinal IBD (21/26). Nine of 16 control dogs underwent colonoscopy in order to access colonic mucosal miRs expression, with serum miR expression analysis being performed in 14/16 dogs.

**Fig. 1:**
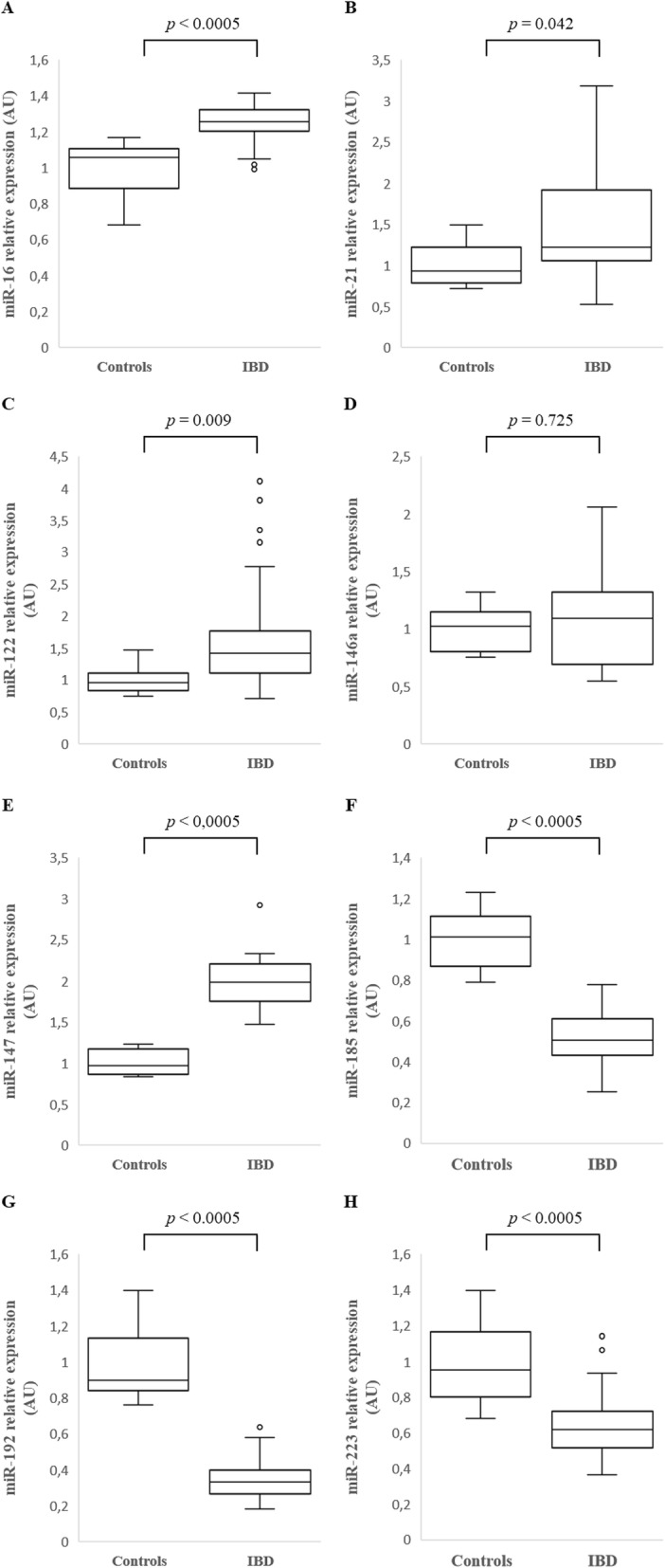
Relative expression of miR-16 (**a**), miR-21 (**b**), miR-122 (**c**), miR-146a (**d**), miR-147 (**e**), miR-185 (**f**), miR-192 (**g**) and miR-223 (**h**) in the colonic mucosa of healthy dogs (Controls) (*n* = 9) and dogs with large intestinal inflammatory bowel disease (IBD) (*n* = 26). Data is presented as median with 25th and 75th quartiles in each box plot. The whiskers indicate the highest and lowest data within 1.5 times the lengths of the quartiles. Statistical significance was defined as *p* < 0.05. AU = arbitrary units (which represent the expression of each miR normalized toU6sn RNA internal control), miR = microRNA

Compared to healthy controls, there was a significantly increased relative expression of the following miRs in the colonic mucosa of dogs with large intestinal IBD: miR-16 (*p* < 0.0005), miR-21 (*p* = 0.042), miR-122 (*p* = 0.009) and miR-147 (*p* < 0.0005), as well as a significantly decreased relative expression of miR-185 (*p* < 0.0005), miR-192 (*p* < 0.0005) and miR-223 (*p* < 0.0005). miR-146a relative expression in the colonic mucosa didn’t show a statistically significant difference between dogs with large intestinal IBD and healthy controls in the colonic mucosa (*p* = 0.725) (Fig. 1).

**Fig. 2:**
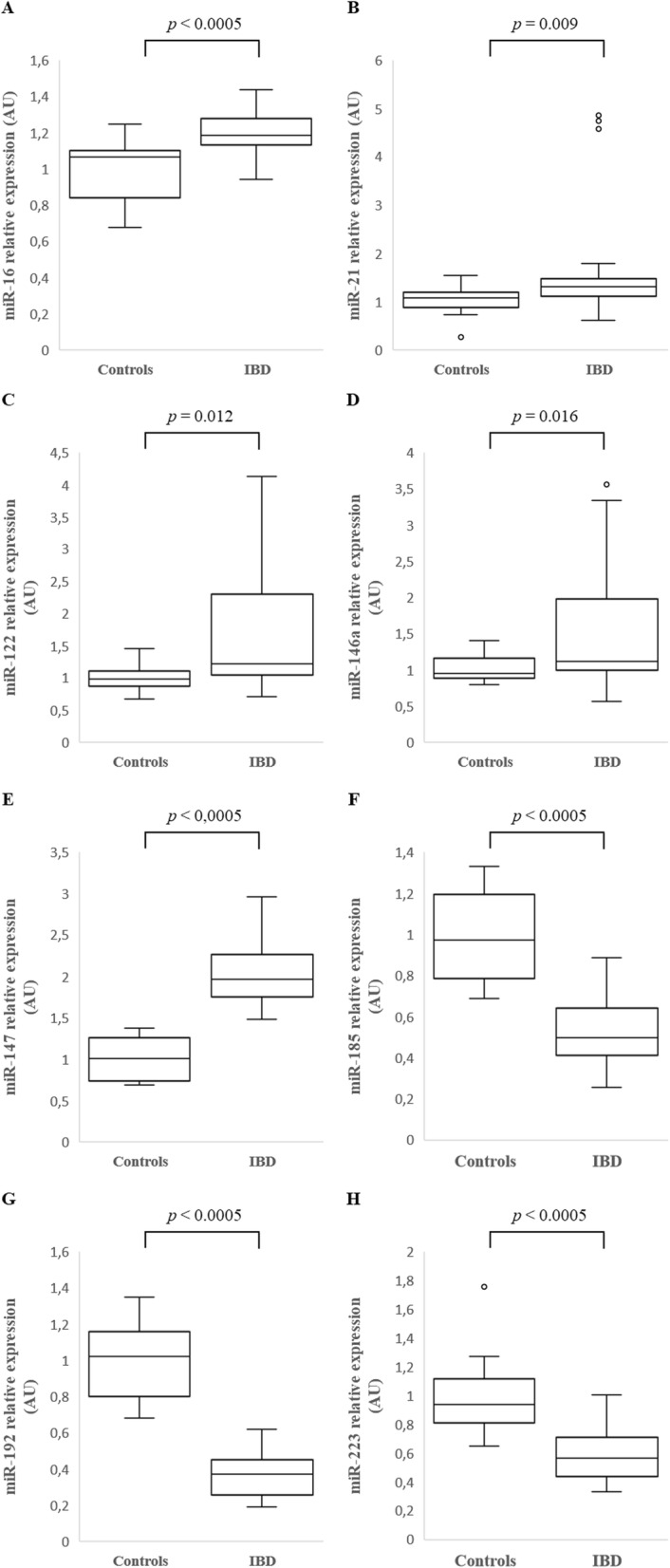
Relative expression of miR-16 (**a**), miR-21 (**b**), miR-122 (**c**), miR-146a (**d**), miR-147 (**e**), miR-185 (**f**), miR-192 (**g**) and miR-223 (**h**) in the serum of healthy dogs (Controls) (*n* = 14) and dogs with large intestinal inflammatory bowel disease (IBD) (*n* = 21). Data is presented as median with 25th and 75th quartiles in each box plot. The whiskers indicate the highest and lowest data within 1.5 times the lengths of the quartiles. Statistical significance was defined as *p* < 0.05. AU = arbitrary units (which represent the expression of each miR normalized toU6sn RNA internal control), miR = microRNA

Compared to healthy controls, there was a statistically significant increased relative expression of the following miRs in the serum of dogs with large intestinal IBD: miR-16 (*p* < 0.0005), miR-21 (*p* = 0.009), miR-122 (*p* = 0.012), miR146a (*p* = 0.016) and miR-147 (*p* < 0.0005), as well as a significantly decreased relative expression of miR-185 (*p* < 0.0005), miR-192 (*p* < 0.0005) and miR-223 (*p* < 0.0005) (Fig. 2).

### Relationship between miRs relative expression, canine chronic enteropathy clinical activity index, colonoscopy score and histopathologic score of dogs with large intestinal IBD

**Fig. 3:**
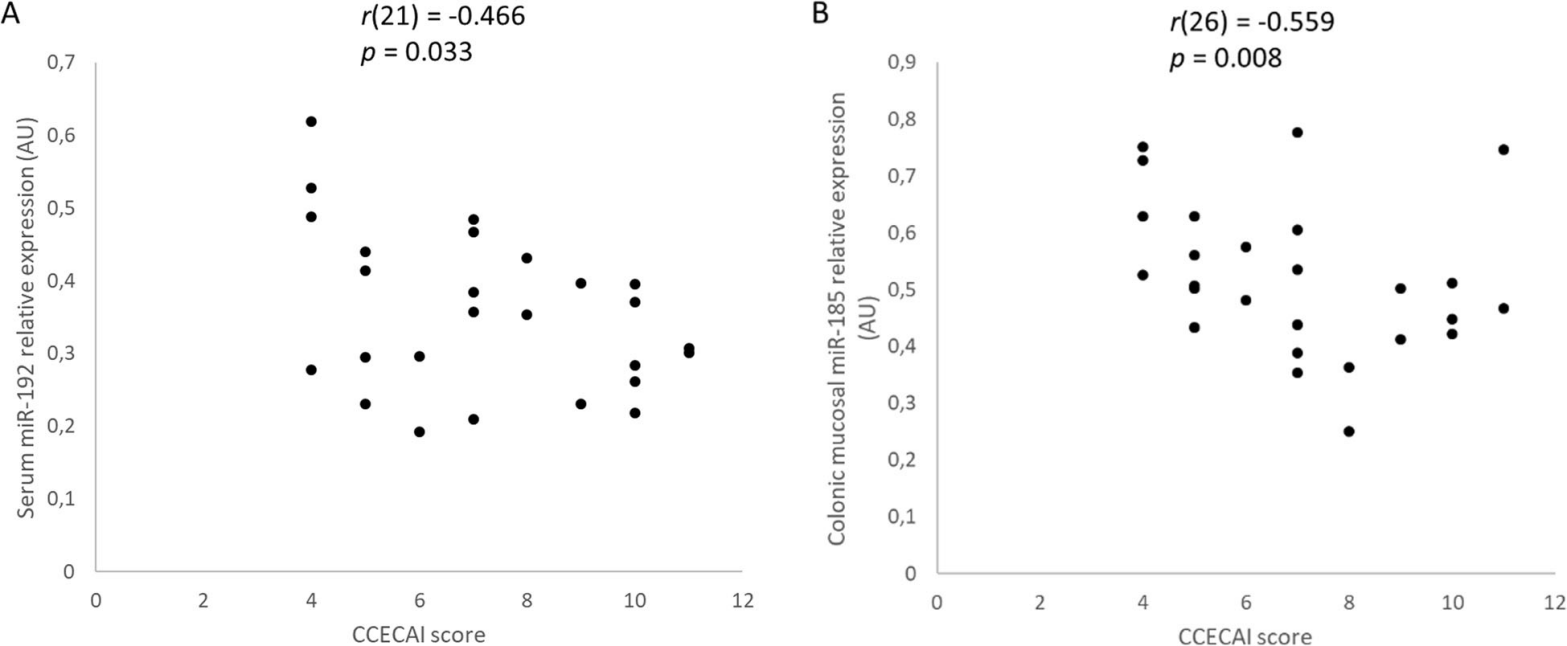
Scatter plots of the relative expression of miR-192 in the serum (*n* = 21) (**a**) and miR-185 in the colonic mucosa (*n* = 26) (**b**), and CCECAI score of dogs with large intestinal inflammatory bowel disease (IBD) as determined by Spearman’s rank correlation. Statistical significance was defined as *p* < 0.05. AU = arbitrary units (which represent the expression of each miR normalized to U6sn RNA internal control), CCECAI = canine chronic enteropathy clinical activity index, miR = microRNA, *r* = Spearman’s rank correlation coefficient

**Fig. 4:**
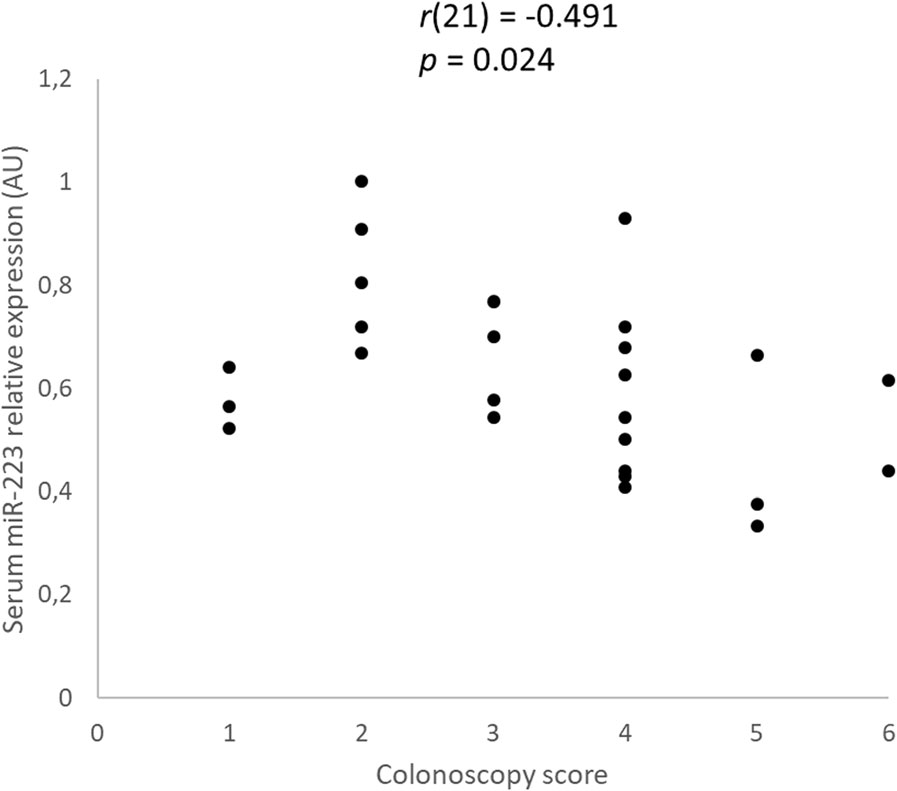
Scatter plot of the relative expression of miR-223 in the serum and colonoscopy score of dogs with large intestinal inflammatory bowel disease (IBD) (*n* = 26) as determined by Spearman’s rank correlation. Statistical significance was defined as *p* < 0.05. AU = arbitrary units (which represent the expression of each miR normalized to U6sn RNA internal control), CCECAI = canine chronic enteropathy clinical activity index, miR = microRNA, *r* = Spearman’s rank correlation coefficient

**Fig. 5:**
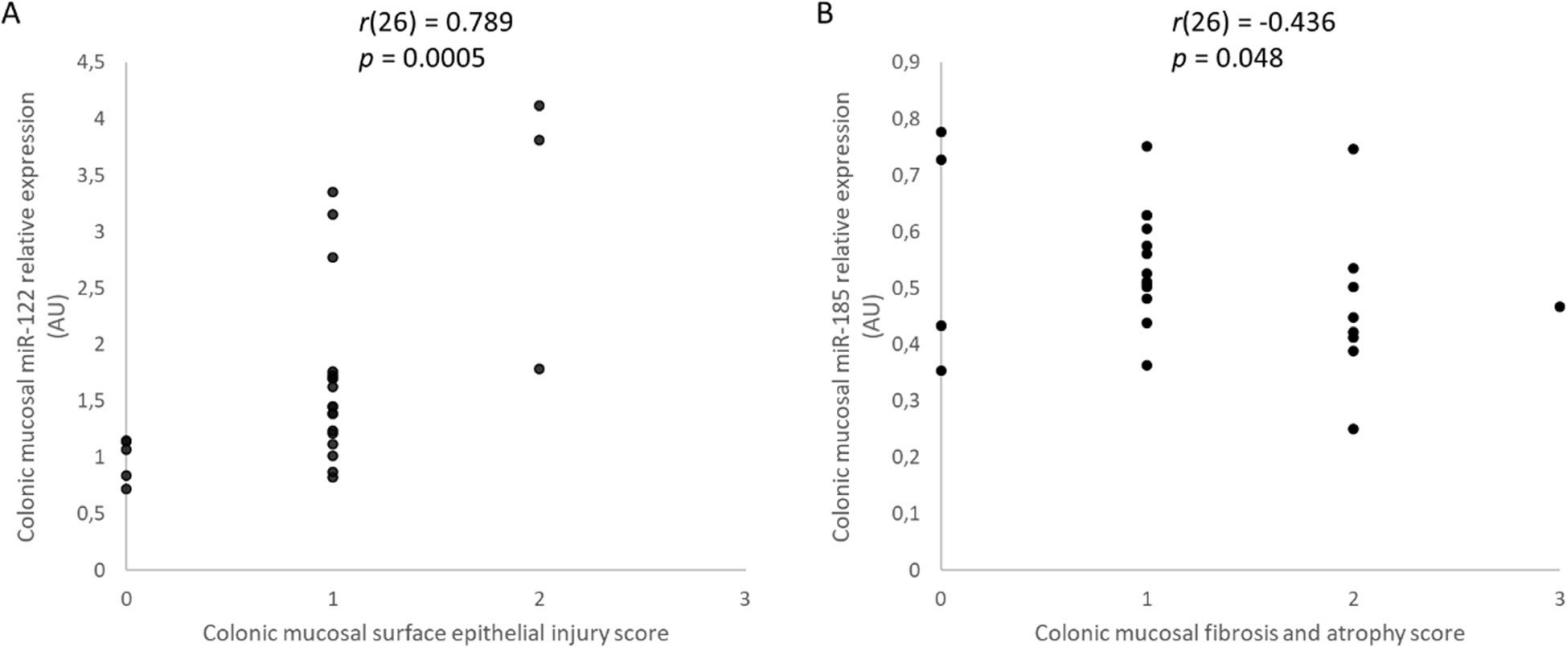
Scatter plots of the relative expression of miR-122 in the colonic mucosa and colonic mucosal epithelial injury score (**a**), and miR-185 in the colonic mucosa and colonic mucosal fibrosis and atrophy score (**b**) of dogs with large intestinal inflammatory bowel disease (IBD) (*n* = 26), as determined by Spearman’s rank correlation. Statistical significance was defined as *p* < 0.05. AU = arbitrary units (which represent the expression of each miR normalized toU6sn RNA internal control), miR = microRNA, *r* = Spearman’s rank correlation coefficient

There was a moderate, statistically significant negative correlation of serum miR-192 (*r* (21) = − 0.466, *p* = 0.033) (Fig. 3 and Additional file 1: Table S1) and of colonic mucosal miR-185 (*r*(26) = − 0.559, *p* = 0.008) (Fig. 3) relative expression with the CCECAI score in dogs with large intestinal IBD. In addition, there was a moderate, statistically significant negative correlation of serum miR-223 relative expression with colonoscopy score (*r*(21) = − 0.491, *p* = 0.024) (Fig. 4 and Additional file 1: Table S1). Analysis of the relationship between colonic mucosal (*n* = 26) and serum (*n* = 21) miRs relative expression and histopathologic score (Table 2) of dogs with large intestinal IBD revealed that there was a strong, statistically significant positive correlation of colonic mucosal miR-122 relative expression with surface epithelial injury (a subscore of the WSAVA index) (*r*(26) = 0.789, *p* = 0.0005) and a moderate, statistically significant negative correlation of colonic mucosal miR-185 expression with mucosal fibrosis and atrophy (other subscores of the WSAVA index) (*r*(26) = − 0.436, *p* = 0.048) (Fig. 5 and Additional file 1: Table S1).

### Relationship between miRs relative expression in the colonic mucosa and serum of dogs with large intestinal IBD

**Fig. 6:**
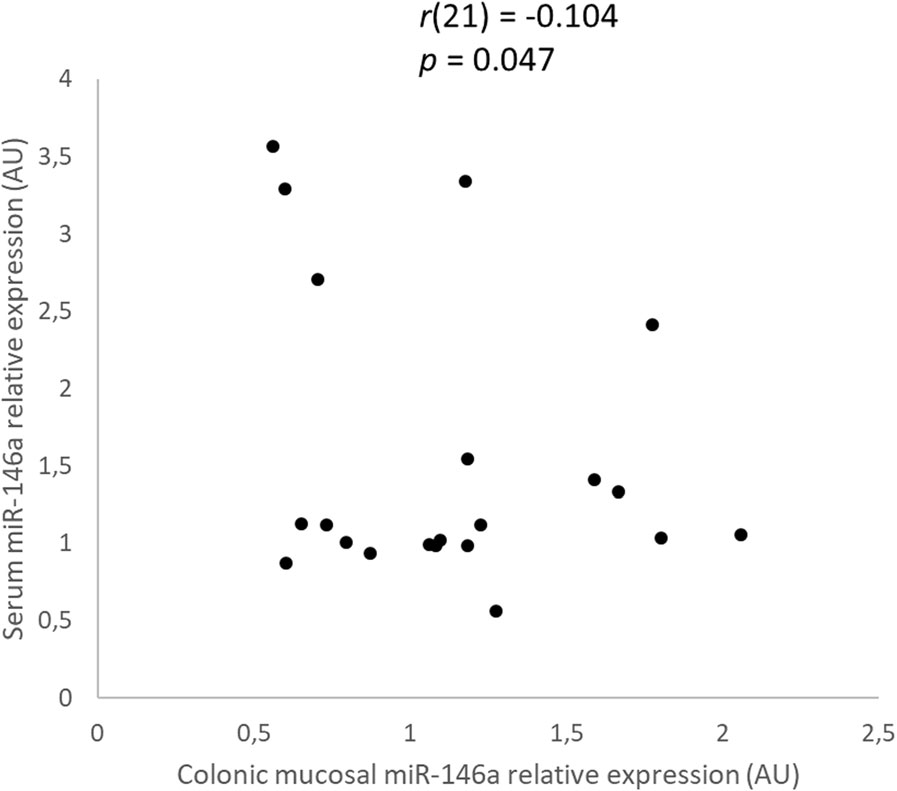
Scatter plots of the relative expression of miR-146a in the colonic mucosa and serum of dogs with large intestinal inflammatory bowel disease (IBD) (*n* = 21) as determined by Spearman’s rank correlation. Statistical significance was defined as *p* < 0.05. AU = arbitrary units (which represent the expression of each miR normalized to U6sn RNA internal control), miR = microRNA, *r* = Spearman’s rank correlation coefficient

There was a weak, statistically significant negative correlation of colonic mucosal with serum miR-146a relative expression (*r*(21) = − 0.104, *p* = 0.047) in dogs with large intestinal IBD (Fig. 6 and Additional file 2: Table S2).

## Discussion

This study evaluated the expression of miR-16, miR-21, miR-122, miR-146a, miR-147, miR-185, miR-192 and miR-223 in the colonic mucosa and serum of dogs with large intestinal IBD compared to healthy controls. It is believed that IBD is the result of the interaction between a dysregulated immune response to luminal (intestinal flora) and/or environmental factors in genetically susceptible individuals. In spite of extensive investigation in animals and humans, the pathogenesis of IBD remains unknown. Evaluation of miRs could offer a potential link between genetic susceptibility, environmental and immunological factors involved in the pathogenesis of IBD and may serve as clinically useful biomarkers for diagnosis and treatment. To our knowledge, this is the first study that has explored changes of miRs expression in canine IBD.

We found a significantly increased relative expression of miR-16, miR-21, miR-122 and miR-147 and a significantly decreased relative expression of miR-185, miR-192 and miR-223 in the colonic mucosa of dogs with IBD compared to healthy controls.

The primary function of miR-16 is to regulate the production of inflammatory mediators and immunity through co-operation with other miRs, targeting to tumor necrosis factor- alpha (TNF-α) [37]. It is involved in the induction of apoptosis by targeting B-cell leukemia-lymphoma 2 (Bcl-2). In humans, miR-16 expression is inversely correlated to Bcl-2 expression in chronic lymphocytic leukemia and negatively regulate Bcl-2 at a posttranscriptional level [38]. This finding agrees with two studies that evaluated apoptosis in canine IBD, where the expression of Bcl-2 was greater in the duodenum and colon of healthy control dogs versus dogs with IBD and greater numbers of Bcl-2 cells were found in the colon of healthy control dogs compared to dogs with IBD. It is therefore possible that this could be the reason for the miR-16 increase in the colonic mucosa that was detected in our study [39, 40].

In mice, miR-21 could be involved in the pathogenesis of IBD through at least 3 different mechanisms targeting epithelial barrier function, apoptosis and fibrosis [41]. Intestinal permeability was greater in wild type compared to miR-21 knockout strain mice with experimental dextran sulfate sodium (DSS) colitis, while knockout mice had also less intestinal epithelial cell apoptosis [42]. Furthermore, mimics of mature miR-21 (synthetic analog of miR-21) transfection resulted in loss of tight junction proteins and increased barrier permeability in an ex vivo colon epithelial cell model [43]. miR-21 has been associated with fibrosis, especially in multiple disease disorders, such as renal fibrosis and cirrhosis [44, 45]. However, miR-21 expression has not been clearly associated with fibrosis in human IBD, a finding that is in agreement with the results of the present study.

miR-122, which is mainly found in the liver, accounts for approximately 70% of all liver miRs, regulates the expression of genes that control cell life cycle [46–48], and is considered to be a specific miR for canine hepatobiliary diseases [26, 27]. Recent studies have investigated the miR-122 expression in human IBD, including pediatric IBD, and tried to clarify its role in the pathogenesis of disease [49–52]. Chen et al. (2013) [51] found that miR-122 targets nucleotide-binding oligomerization domain-2 (NOD2), increasing the anti- inflammatory (IL-4 and IL-10) and decreasing pro-inflammatory cytokines, TNF-α and Interferon-γ (IFN-γ), in Crohn’s disease (CD). In another study investigating pediatric CD, there was no difference in the expression of miR-122 in the inflamed mucosa compared to healthy duodenal mucosa of children [50]; however, increased expression of miR-122 was found in the macroscopically normal colonic mucosa of children with CD compared to healthy controls indicating that it may be a marker of reduced intestinal epithelial cell injury [49]. The overexpression of miR-122 in the colonic mucosa of our dogs with IBD, compared to healthy dogs, along with the strongly positive correlation of miR-122 relative expression in the colonic mucosa with the score of epithelial cell injury in the colon, may be attributed to differences in disease pathogenesis, type of infiltrating cells, as well as disease severity and prognosis between canine IBD and pediatric CD.

miR-146a influences numerous biological functions that play important roles in immune-mediated diseases (inflammatory response, intracellular signaling cascades, regulation of cytokine production, and response to bacteria) [53–56]. miR-146a is involved in innate immunity by regulating the acute inflammatory response after pathogen recognition by toll-like receptors (TLR), by downregulating the expression of TNF receptor-associated factor 6 and interleukin-1 receptor-associated kinase 1 [57]. miR-146a controls the adaptive immune response by influencing regulatory T cells (Tregs), thereby controlling T helper 1 cell (Th1) responses [58, 59]. In the intestine miR-146a is involved in regulating the expansion of certain intestinal T cell subtypes: T helper 17 cells (Th17), Tregs, and T follicular helper cells (Tfh) [60]. miR-146a also has a role in protecting the barrier function, as its deficiency was accompanied with a more resistant phenotype during experimental murine colitis [60]. Several studies have found increased expression of miR-146a in adults and children with CD and ulcerative colitis (UC), in both macroscopically inflamed and intact mucosa [49, 50, 61–64].

Colon and ileum of healthy dogs are the primary tissues of miR-147 expression [25]. miR-147 expression has not been studied in human IBD or murine models of the disease. Liu et al. [65] found that miR-147 is associated with inflammation, as it acts as a negative regulator of TLR-induced signaling in murine macrophages. miR-147 had a decreasing effect on inflammatory cytokine expression, while silencing of miR-147 increased the expression of inflammatory cytokines in macrophages stimulated with ligands of TLR2, TLR3 and TLR4. Increased duodenal and colonic mucosal expression of TLR2, TLR4 and TLR9 and downregulated expression of TLR5 has been reported in dogs with IBD, but without correlation with either histologic severity of inflammation or response to treatment [66–68]. Taking into account all this information, authors suggest that miR-147 could be implicated in canine IBD pathogenesis, especially when the large intestine is involved.

Currently there is little evidence for the role of miR-185 in human IBD. miR-185 is generally regarded as a regulator involved in the biological processes of neoplastic cells and neurological disorders [69–74]. miR-185 expression investigation was selected because there are data supporting its usefulness as a CD specific diagnostic marker, both in adults and children [62, 75]. In contrast to human studies, we found decreased relative expression in the colonic mucosa of dogs with IBD compared to healthy controls. Furthermore, we found a negative correlation of miR-185 relative expression in the colonic mucosa of dogs with IBD with CCECAI score and scores of mucosal fibrosis and atrophy. Expression change of miR-185 in human (increased) and canine (decreased) IBD suggest that it is involved in disease pathogenesis, however its exact role and mechanism of gene regulation remain to be elucidated with further studies.

miR-192 is predominantly expressed in the colonic epithelial cells in humans and shows reduced levels in active UC [76]. miR-192 is regulating colonic mucosal chemokine expression. Epithelial miR-192 directly regulates the expression of macrophage inflammatory peptide-2 alpha (MIP-2α), while the induction of MIP-2α simultaneously decreases miR-192 expression [76]. Moreover, miR-192 significantly alters NOD2 mRNA and protein expression, and miR-192 mimic significantly reduced nuclear factor- kB (NF-kB) phosphorylation and expression of C-X-C motif ligand 8 (CXCL8/IL-8) and C-X-C motif ligand 3 (CXCL3) [77]. Maeda et al. [78] reported the up-regulation of CXCL8/IL-8 in the duodenal mucosa in canine IBD. These results may suggest that miR-192 is a key miR in the pathogenesis of IBD and regulation of intestinal epithelial cell innate immune response, and perhaps this is the reason of its reduced relative expression in the colonic mucosa of our dogs with large intestinal IBD.

miR-223 modulates innate and adaptive immune mechanisms by regulating families of immune-related target mRNAs in multiple inflammatory and autoimmune processes in humans, including IBD [23, 79–81]. Its expression has been extensively studied in humans with IBD. Increased expression of miR-223 in the mucosa, serum and feces has been consistently found in CD and UC patients, including pediatric patients [31, 62, 82, 83]. miR-223 is highly expressed in neutrophilic granulocytes [84]. Contrary to human studies, we found decreased expression of miR-223 in dogs with large intestinal IBD compared to healthy controls perhaps due to the different predominating cell population in human and canine IBD.

miRs characteristics, support their measurement in circulation as potential, non-invasive and sensitive biomarkers for the diagnosis and determination of progression of the disease. We found a significantly increased relative expression of miR-16, miR-21, miR-122, miR-146a and miR-147 and a significantly decreased relative expression of miR-185, miR-192 and miR-223 in the serum of dogs with large intestinal IBD compared to healthy controls. Their relative expression profiles were similar to those in the colonic mucosa, except of miR-146a which showed a negative but weak correlation, with no clinical correlation. However, we did not find a significant correlation between their expression in the colonic mucosa and serum. On the other hand, in human IBD various studies have compared miRs expression profiles between tissue and blood and it was concluded that they do not necessarily parallel expression in different sites [24, 30, 31, 62, 64, 85]. Two different studies found increased expression of miR-19a in both colonic mucosa and serum of CD patients [30, 62]. Moreover the expression of miR-29a, miR-106a, miR-126, miR-191, and miR-200c in blood samples of CD patients and the expression of miR-21 and miR-155 in blood samples of UC patients reflected the expression of correlated diseased tissues [28, 30, 31, 62, 76]. On the other hand, miR-188-5p was found to be downregulated in colon tissue of active and inactive UC patients [62], inversely of the upregulation observed in platelets of inactive UC patients [85]. In addition, miR-19b was found downregulated in sigmoid colon mucosa samples of CD patients, while in separate study in serum from 46 pediatric CD patients showed increased expression [30, 31].

We found a statistically significant moderately negative correlation of serum miR-192 relative expression with CCECAI score of dogs with large intestinal IBD. In addition, we found a statistically significant moderately negative correlation of serum miR-223 relative expression with endoscopic score. Recent studies have evaluated the association between the endoscopic examination score and long-term outcome in dogs with chronic enteropathies or have utilized endoscopic evaluation to assess mucosal healing pre- and post-treatment in dogs with lymphocytic-plasmacytic gastroenteritis [86, 87]. The vast majority of dogs with IBD display endoscopic improvement after successful treatment. Thus, miR-192 and miR-223 could be useful for the evaluation of disease severity, prediction of flares, response to treatment and prognosis. However, more studies are needed to assess their clinical utility.

There are some limitations of the current study. Owners’ denial to participate in the study was the main reason of the relatively limited number of IBD cases. Furthermore, we included only large intestinal IBD cases. miRs expressions in canine IBD affecting different segments of the GI tract or with other predominant cell type infiltration could be different than those observed in our study. Finally, miRs selection of our study was based on data from human literature without previously performing microarray analysis. Microarray analysis of all miRs would have been ideal, but is costly and was beyond the scope of the current study. This means that some key miRs in canine IBD may have been missed.

## Conclusions

In conclusion, this is the first study describing the expression of a miRs panel in canine IBD. We demonstrate that dogs with large intestinal IBD have significant changes in miRs expression levels in the colonic mucosa and the serum. It was found that miR-16, miR-21, miR-122 and miR-147 expression had significantly increased relative expression, while miR-185, miR-192 and miR-223 expression were significantly decreased in the colonic mucosa and serum of dogs with large intestinal IBD compared to healthy controls. miR-146a expression increased significantly only in the serum of dogs with large intestinal IBD. These changes suggest that miRs are involved in the pathogenesis of IBD. Furthermore, serum miR-192 and miR-223 relative expression correlated to disease activity and endoscopic score, respectively. The results of this study may become the basis for further studies which will investigate miRs expression in canine IBD and other chronic enteropathies at diagnosis and in response to treatment.

## Methods

### Dogs and study design

Dogs with large intestinal IBD: 26 dogs presented or referred to Companion Animal Clinic, School of Veterinary Medicine, Aristotle University of Thessaloniki for investigation of large intestinal disease and were diagnosed with IBD. Inclusion criteria in order the dogs to be enrolled in the study were (1) history of chronic (> 3 weeks) large intestinal disease (large intestinal diarrhea with mucus, hematochezia, tenesmus, and/or increased frequency of defecation) without any identifiable underlying cause, (2) histopathologic evidence of intestinal inflammatory cellular infiltration and (3) minor or no response to a 3-week dietary trial (novel or hydrolyzed protein diets). Exclusion criteria were (1) the presence of a concurrent disease and (2) administration of any medication for a 2-week period prior to presentation or referral. The experimental protocol was reviewed and approved by the ethics committee of the School of Veterinary Medicine, Aristotle University of Thessaloniki (58/ 29-9-2015). Owners provided informed written consent for enrollment of their dog to the study. All dogs underwent physical examination and diagnostic investigation to exclude other known causes of large intestinal diarrhea, i.e. complete blood count, routine serum biochemistry, serum folate and cobalamin concentrations, serum cPLI concentration (SNAP® cPL Test, IDEXX Laboratories, Westbrook, ME, USA), detection of Anti-Leishmania infantum antibodies in serum (Leishmania SNAP® Test, IDEXX Laboratories, Westbrook, ME, USA), detection of Anti-Anaplasma phagocytophilum/Anaplasma platys, − Borrelia burgdorferi, and - Ehrlichia canis antibodies and the Dirofilaria immitis antigen in serum (4DX SNAP® Test, IDEXX Laboratories, Westbrook, ME, USA), ACTH stimulation test, urinalysis, abdominal radiographic and ultrasonographic examination, parasitological and cytological examination of feces and SNAP® Giardia Test (IDEXX, Laboratories, Westbrook, ME, USA) for the detection of Giardia-specific cyst wall antigen in feces. Clinical disease activity was recorded for all dogs using the CCECAI [86].

Healthy dogs (controls): 16 healthy adult dogs with no GI signs for at least 6 months prior to diagnostic evaluation were included in this study. The health status of the dogs was estimated by physical examination, complete blood count, routine serum biochemistry, urinalysis, and parasitological and cytological examination of feces, as well as determination of Anti-Leishmania infantum, Anti-Anaplasma phagocytophilum/Anaplasma platys, − Borrelia burgdorferi, and - Ehrlichia canis antibodies and Dirofilaria immitis antigen in serum. Colonoscopy and serum miR expression analysis was also performed. All dog owners provided informed written consent permitting enrollment of their dog into the study.

### Sample collection

Dogs underwent colonoscopy under general anesthesia after recommended preparation. Colonoscopies were performed using a CF-140 L flexible video endoscope (Olympus®, Hamburg, Germany) and biopsy tissue specimens were collected from each of the 3 segments of the colon (ascending, transverse, and descending) using single-use multiple sample biopsy forceps (Multibite™; Boston Scientific, Marlborough, MA, USA). More specifically, we collected totally 13 biopsy specimens, 3 from each segment for histopathologic examination and 4 for miR expression analysis were collected. Endoscopic findings were graded using the quantitative assessment of mucosal appearance for endoscopic activity for canine IBD according to the system proposed by Slovak et al. (2015) [88]. Three endoscopic parameters (friability, granularity and erosions) were scored (0, absent; 1, mild-to-moderate; 2, moderate-to-severe) in each case; the maximum total score was that of 6.

Venous blood samples for miR determination were obtained from all study dogs. Serum was separated within 30 min and stored at − 80 °C until miR analysis.

Biopsy specimens were fixed in 10% neutral buffered formalin for 48 h, paraffin- embedded, sectioned at 3–4 μm, stained with H&E, examined histologically and graded according to the World Small Animal Veterinary Association (WSAVA) GI histopathologic guidelines [89]. Histopathologic examination of biopsy specimens was performed by a single pathologist (GDB) blinded as to the health status of the dog.

### RNA isolation, complementary DNA synthesis, and quantification

Total RNA from colonic mucosa biopsies and serum was isolated using TriZol method. The expression patterns of selected miRs and a housekeeping gene, U6sn, were quantitatively assayed using reverse transcription (RT) and real-time reverse transcription polymerase chain reaction (RT-PCR). Stem-loop complementary DNAs (cDNAs) were synthesized using looped reverse transcription primers specific for each miR. In real-time PCR assays, forward primers specific for each cDNA and a reverse primer universal for all cDNAs were used. The expression of miR-16, miR-21, miR-122, miR146a, miR147, miR-185, miR-192 and miR-223 were examined. All samples were analyzed twice to confirm reproducibility.

### Data normalization

miR-16, miR-21, miR-122, miR-146a, miR-147, miR-185, miR-192 and miR-223 and U6sn were reliably amplified in tested samples. Amplified miRs showed specific melting temperature, confirming the accuracy and specificity of the method used. Real-time quantitative RT-PCR was conducted on an ABI Prism 7700 apparatus (Applied Biosystems™, Foster City, CA, USA). Data were analyzed with the ABI Prism 7700 SDS software (Applied Biosystems™, Foster City, CA, USA). The expression of each miR was normalized to U6sn RNA internal control. The levels of miRs expression were normalized after subtracting the Ct value of the U6sn RNA internal control from that of each miR Ct value for samples (ΔCt = |C_tmiR_ (samples) − C_tU6sn_|). The relative mRNA expression level of each miR (in arbitrary units- AU) was calculated by dividing the expression level by the mean value in control samples; the latter was considered equal to 1.

### Statistical analysis

Statistical analysis was performed using IBM SPSS 19 software program (USA, Chicago, Illinois). Mann–Whitney U test or T- test was performed to compare differences in miRs levels between dogs with large intestinal IBD and healthy control dogs. Correlations of miRs expression with CCECAI score, canine IBD quantitative endoscopic activity and histopathologic score, as well as correlation of colonic mucosal miRs expression with serum miRs expression of dogs with large intestinal IBD were also statistically analyzed using Spearman’s correlation coefficient test. *P* values of < 0.05 were considered significant.

## Supplementary information

**Additional file 1: Table S1.** Correlations of the relative expression of miR-16, miR-21, miR-122, miR-146a, miR-147, miR-185, miR-192 and miR-223 in the serum (*n* = 21) and the colonic mucosa (*n* = 26) with inflammatory and morphologic histopathologic features score according to the World Small Animal Veterinary Association (WSAVA) GI Standardization Group guidelines in defining inflammation involving the colon of dogs with large intestinal inflammatory bowel disease (IBD). LP = lamina propria, miR = microRNA.

**Additional file 2: Table S2.** Correlations of the relative expression of miR-16, miR-21, miR-122, miR-146a, miR-147, miR-185, miR-192 and miR-223 in the serum (*n* = 21) and the colonic mucosa (*n* = 26) with CCECAI and colonoscopy score of dogs with large intestinal inflammatory bowel disease (IBD). CCECAI = canine chronic enteropathy clinical activity index, miR = microRNA.

## Data Availability

The datasets used and/or analyzed during the current study are available from the corresponding author on reasonable request.

## Abbreviations

Bcl-2: B- cell leukemia- lymphoma 2
CCECAI: Canine chronic enteropathy clinical activity index
CD: Crohn’s disease
cDNA: Complementary DNA
CIBDAI: Canine inflammatory bowel disease activity index
CXCL3: C-X-C motif ligand 3
CXCL8: C-X-C motif ligand 8
DSS: Dextran sulfate sodium
GI: Gastrointestinal
IBD: Inflammatory bowel disease
IFN-γ: Interferon gamma
MIP-2a: Macrophage inflammatory peptide-2 alpha
miR: microRNAs
mRNA: MessengerRNA
NF-kB: Nuclear factor- kB
PCR: Polymerace chain reaction
RT-PCR: Reverse transcription polymerase chain reaction
Tfc: T follicular helper cells
Tfh: T follicular helper cells
Th1: T helper 1 cells
Th17: T helper 17 cells
TLR: Toll-like receptor
TNF-α: Tumor necrosis factor- alpha
Treg: Regulatory T cells
UC: Ulcerative colitis

## References

[1] Allenspach K (2011). Clinical immunology and immunopathology of the canine and feline intestine. Vet Clin North Am - Small Anim Pract.

[2] Gaschen FP, Allenspach K, Washabau RJ, Day M (2013). Large intestine- inflammation. Canine & Feline Gastroenterology.

[3] Bartel DP (2009). MicroRNAs: target recognition and regulatory functions. Cell.

[4] Liu W, Mo SY, Zhu WY (2007). Impact of tiny miRNAs on cancers. World J Gastroenterol.

[5] Meunier J, Lemoine F, Soumillon M, Liechti A, Weier M, Guschanski K (2013). Birth and expression evolution of mammalian microRNA genes. Genome Res.

[6] Lagos-Quintana M, Rauhut R, Lendeckel W, Tuschl T (2001). Identification of novel genes coding for small expressed RNAs. Science.

[7] Ambros V (2004). The functions of animal microRNAs. Nature..

[8] Gazouli M (2015). Circulating microRNAs in disease diagnostics and their potential biological relevance. Experimentia.

[9] Koukos G, Polytarchou C, Kaplan J, Morley-Fletcher A, Gras-miralles B, Kokkotou E (2015). MicroRNA-124 regulates STAT3 expression and is downregulated in colon tissues of pediatric patients with ulcerative colitis. Gastroenterology.

[10] Chen X, Ba Y, Ma L, Cai X, Yin Y, Wang K (2008). Characterization of microRNAs in serum: a novel class of biomarkers for diagnosis of cancer and other diseases. Cell Res.

[11] Mitchell PS, Parkin RK, Kroh EM, Fritz BR, Wyman SK, Pogosova-Agadjanyan EL (2008). Circulating microRNAs as stable blood-based markers for cancer detection. Proc Natl Acad Sci.

[12] Lawrie CH, Gal S, Dunlop HM, Pushkaran B, Liggins AP, Pulford K (2008). Detection of elevated levels of tumour-associated microRNAs in serum of patients with diffuse large B-cell lymphoma. Br J Haematol.

[13] Weber JA, Baxter DH, Zhang S, Huang DY, Huang KH, Lee MJ (2016). The microRNA spectrum in 12 body fluids. Clin Chem.

[14] Turchinovich A, Weiz L, Langheinz A, Burwinkel B (2011). Characterization of extracellular circulating microRNA. Nucleic Acids Res.

[15] Brase JC, Wuttig D, Kuner R, Sültmann H (2010). Serum microRNAs as non-invasive biomarkers for cancer. Mol Cancer.

[16] Link A, Balaguer F, Shen Y, Nagasaka T, Lozano JJ, Boland CR (2010). Fecal microRNAs as novel biomarkers for colon cancer screening. Cancer Epidemiol Biomark Prev.

[17] Lorenzen JM, Haller H, Thum T (2011). MicroRNAs as mediators and therapeutic targets in chronic kidney disease. Nat Rev Nephrol.

[18] Calin GA, Croce CM (2006). MicroRNA signatures in human cancers. Nat Rev Cancer.

[19] Kerr TA, Korenblat KM, Davidson NO (2011). MicroRNAs and liver disease. Transl Res.

[20] Pekow J, Kwon J (2011). MicroRNAs in inflammatory bowel disease. Inflamm Bowel Dis.

[21] Rybaczyk L, Rozmiarek A, Circle K, Grants I, Needleman B, Wunderlich JE (2009). New bioinformatics approach to analyze gene expressions and signaling pathways reveals unique purine gene dysregulation profiles that distinguish between CD and UC. Inflamm Bowel Dis.

[22] Pauley KM, Satoh M, Chan AL, Bubb MR, Reeves WH, Chan EK (2008). Upregulated miR-146a expression in peripheral blood mononuclear cells from rheumatoid arthritis patients. Arthritis Res Ther.

[23] Sonkoly E, Wei T, Janson PCJ, Sääf A, Lundeberg L, Tengvall-Linder M (2007). MicroRNAs: novel regulators involved in the pathogenesis of psoriasis?. PLoS One.

[24] Iborra M, Bernuzzi F, Correale C, Vetrano S, Fiorino G, Beltrán B (2013). Identification of serum and tissue micro-RNA expression profiles in different stages of inflammatory bowel disease. Clin Exp Immunol.

[25] Koenig EM, Fisher C, Bernard H, Wolenski FS, Gerrein J, Carsillo M (2016). The beagle dog microRNA tissue atlas: identifying translatable biomarkers of organ toxicity. BMC Genomics.

[26] Dirksen K, Verzijl T, Grinwis GC, Favier RP, Penning LC, Burgener IA (2016). Use of serum microRNAs as biomarker for hepatobiliary diseases in dogs. J Vet Intern Med.

[27] Oosthuyzen W, Ten Berg PWL, Francis B, Campbell S, Macklin V, Milne E (2018). Sensitivity and specificity of microRNA-122 for liver disease in dogs. J Vet Intern Med.

[28] Paraskevi A, Theodoropoulos G, Papaconstantinou I, Mantzaris G, Nikiteas N, Gazouli M (2012). Circulating microRNA in inflammatory bowel disease. J Crohn's Colitis.

[29] Jensen MD, Andersen RF, Christensen H, Nathan T, Kjeldsen J, Madsen JS (2015). Circulating microRNAs as biomarkers of adult Crohn’s disease. Eur J Gastroenterol Hepatol.

[30] Zahm AM, Thayu M, Hand NJ, Horner A, Leonard MB, Friedman JR (2011). Circulating microRNA is a biomarker of pediatric crohn disease. J Pediatr Gastroenterol Nutr.

[31] Wu F, Zhang S, Dassopoulos T, Harris ML, Bayless TM, Meltzer SJ (2010). Identification of microRNAs associated with ileal and colonic Crohn’s disease. Inflamm Bowel Dis.

[32] Heilmann RM, Allenspach K (2017). Pattern-recognition receptors: signaling pathways and dysregulation in canine chronic enteropathies—brief review. J Vet Diagnostic Investig.

[33] Jergens AE, Sonea IM, O’Connor AM, Kauffman LK, Grozdanic SD, Ackermann MR (2009). Intestinal cytokine mRNA expression in canine inflammatory bowel disease: a meta-analysis with critical appraisal. Comp Med.

[34] Papaconstantinou I, Stamatis K, Tzathas C, Vassiliou I, Giokas G, Gazouli M (2013). The role of variations within microRNA in inflammatory bowel disease. Eur J Gastroenterol Hepatol.

[35] Kanneganti TD (2017). Inflammatory bowel disease and the NLRP3 inflammasome. J Med.

[36] Tili E, Michaille JJ, Piurowski V, Rigot B, Croce CM (2017). MicroRNAs in intestinal barrier function, inflammatory bowel disease and related cancers — their effects and therapeutic potentials. Curr Opin Pharmacol.

[37] Tomankova T, Petrek M, Gallo J, Kriegova E (2012). MicroRNAs: emerging regulators of immune-mediated diseases. Scand J Immunol.

[38] Cimmino A, Calin GA, Fabbri M, Iorio MV, Ferracin M, Shimizu M (2005). miR-15 and miR-16 induce apoptosis by targeting Bcl-2. Proc Natl Acad Sci.

[39] Dandrieux JR, Doherr MG, Kano R, Zurbriggen A, Burgener IA (2008). Evaluation of lymphocyte apoptosis in dogs with inflammatory bowel disease. Am J V.

[40] Jergens A, Young J, Moore D, Wang C, Hostetter J, Augustine L (2014). Bcl-2/Caspase 3 mucosal imbalance favors T cell resistance to apoptosis in dogs with inflammatory bowel disease. Vet Immunol Immunopathol.

[41] Fisher K (2015). MicroRNA in inflammatory bowel disease: translational research and clinical implication. World J Gastroenterol.

[42] Shi C, Liang Y, Yang J, Xia Y, Chen H, Han H (2013). MicroRNA-21 knockout improve the survival rate in DSS induced fatal colitis through protecting against inflammation and tissue injury. PLoS One.

[43] Yang Y, Ma Y, Shi C, Chen H, Zhang H, Chen N (2013). Overexpression of miR-21 in patients with ulcerative colitis impairs intestinal epithelial barrier function through targeting the rho GTPase RhoB. Biochem Biophys Res Commun.

[44] Zarjou A, Yang S, Abraham E, Agarwal A, Liu G (2011). Identification of a microRNA signature in renal fibrosis: role of miR-21. AJP Ren Physiol.

[45] Zhao J, Tang N, Wu K, Dai W, Ye C, Shi J (2014). MiR-21 simultaneously regulates ERK1 signaling in HSC activation and hepatocyte EMT in hepatic fibrosis. PLoS One.

[46] Boutz DR, Collins PJ, Suresh U, Lu M, Ramírez CM, Fernández-Hernando C (2011). Two-tiered approach identifies a network of cancer and liver disease-related genes regulated by miR-122. J Biol Chem.

[47] Lagos-Quintana M, Rauhut R, Yalcin A, Meyer J, Lendeckel W, Tuschl T (2002). Identification of tissue-specific microRNAs from mouse. Curr Biol.

[48] Chang J, Nicolas E, Marks D, Sander C, Lerro A, Buendia MA (2004). miR-122, a mammalian liver-specific microRNA, is processed from hcr mRNA and may downregulate the high affinity cationic amino acid transporter CAT-1. RNA Biol.

[49] Béres NJ, Szabó D, Kocsis D, Szucs D, Kiss Z, Müller KE (2016). Role of altered expression of MIR-146a, MIR-155, and MIR-122 in pediatric patients with inflammatory bowel disease. Inflamm Bowel Dis.

[50] Szűcs D, Béres NJ, Rokonay R, Boros K, Borka K, Kiss Z (2016). Increased duodenal expression of miR-146a and −155 in pediatric Crohn’s disease. World J Gastroenterol.

[51] Chen Y, Wang C, Liu Y, Tang L, Zheng M, Xu C (2013). MiR-122 targets NOD2 to decrease intestinal epithelial cell injury in Crohn’s disease. Biochem Biophys Res Commun.

[52] Kanaan Z, Rai SN, Eichenberger MR, Dworkin AM, Weller C, Cohen E (2015). Differential microRNA expression tracks neoplastic progression in inflammatory bowel disease-associated colorectal cancer. Hum Mutat.

[53] Iborra M, Bernuzzi F, Invernizzi P, Danese S (2012). MicroRNAs in autoimmunity and inflammatory bowel disease: crucial regulators in immune response. Autoimmun Rev.

[54] Chen W-X, Ren L-H, Shi R-H (2014). Implication of miRNAs for inflammatory bowel disease treatment: systematic review. World J Gastrointest Pathophysiol.

[55] Dalal SR, Kwon JH (2010). The role of microRNA in inflammatory bowel disease. Gastroenterol Hepatol.

[56] Raisch J, Darfeuille-Michaud A, Nguyen HTT (2013). Role of microRNAs in the immune system, inflammation and cancer. World J Gastroenterol.

[57] Taganov KD, Boldin MP, Chang K, Baltimore D (2006). NFkB-dependent induction of microRNA miR-146, an inhibitor targeted to signaling proteins of innate immune responses. Proc Natl Acad Sci U S A.

[58] Yang L, Boldin MP, Yu Y, Liu CS, Ea C-K, Ramakrishnan P (2012). *miR-146a* controls the resolution of T cell responses in mice. J Exp Med.

[59] Lu LF, Boldin MP, Chaudhry A, Lin LL, Taganov KD, Hanada T (2010). Function of miR-146a in controlling Treg cell-mediated regulation of Th1 responses. Cell..

[60] Runtsch MC, Hu R, Alexander M, Wallace J, Petersen C, Valentine JF (2015). MicroRNA-146a constrains multiple parameters of intestinal immunity and increases susceptibility to DSS colitis. Oncotarget.

[61] Lin J, Welker NC, Zhao Z, Li Y, Zhang J, Reuss SA (2014). Novel specific microRNA biomarkers in idiopathic inflammatory bowel disease unrelated to disease activity. Mod Pathol.

[62] Fasseu M, Tréton X, Guichard C, Pedruzzi E, Cazals-Hatem D, Richard C (2010). Identification of restricted subsets of mature microRNA abnormally expressed in inactive colonic mucosa of patients with inflammatory bowel disease. PLoS One.

[63] Schaefer JS, Attumi T, Opekun AR, Abraham B, Hou J, Shelby H (2015). MicroRNA signatures differentiate Crohn’s disease from ulcerative colitis. BMC Immunol.

[64] Zahm AM, Hand NJ, Tsoucas DM, Le Guen CL, Baldassano RN, Friedman JR (2014). Rectal microRNAs are perturbed in pediatric inflammatory bowel disease of the colon. J Crohns Colitis.

[65] Liu G, Friggeri A, Yang Y, Park Y-J, Tsuruta Y, Abraham E (2009). miR-147, a microRNA that is induced upon toll-like receptor stimulation, regulates murine macrophage inflammatory responses. Proc Natl Acad Sci U S A.

[66] Allenspach K, Smith K, McNeill FM, Hendricks A, Elson-Riggins J, House A (2010). Evaluation of mucosal bacteria and histopathology, clinical disease activity and expression of toll-like receptors in German shepherd dogs with chronic enteropathies. Vet Microbiol.

[67] McMahon LA, Catchpole B, Elson-Riggins J, Riddle A, Smith K, House AK (2010). Expression of toll-like receptor 2 in duodenal biopsies from dogs with inflammatory bowel disease is associated with severity of disease. Vet Immunol Immunopathol.

[68] Burgener IA, Konig K, Allenspach K, Sauter SN, Boisclair J, Doherr MG (2008). Upregulation of toll-like receptors in chronic enteropathies in dogs. J Vet Intern Med.

[69] Ma X, Shen D, Li H, Zhang Y, Lv X, Huang Q, Gao Y (2015). MicroRNA-185 inhibits cell proliferation and induces cell apoptosis by targeting VEGFA directly in von Hippel-Lindau – inactivated clear cell renal cell carcinoma. Urol Oncol Semin Orig Investig.

[70] Wang R, Tian S, Wang H, Chu D, Cao J, Xia H (2014). MiR-185 is involved in human breast carcinogenesis by targeting Vegfa. FEBS Lett.

[71] Tang H, Wang Z, Liu X, Liu Q, Xu G, Li G (2012). LRRC4 inhibits glioma cell growth and invasion through a miR-185- dependent pathway. Curr Cancer Drug Targets.

[72] Qadir XV, Han C, Lu D, Zhang J, Wu T (2014). MicroRNA-185 inhibits hepatocellular carcinoma growth by targeting the DNMT1 / PTEN / Akt pathway. Am J Pathol.

[73] Li Q, Wang J, He Y, Feng C, Zhang X, Sheng J (2014). MicroRNA-185 regulates chemotherapeutic sensitivity in gastric cancer by targeting apoptosis repressor with caspase recruitment domain. Cell Death Dis.

[74] Ding H, Huang Z, Chen M, Wang C, Chen X, Chen J (2015). Identification of a panel of fi ve serum miRNAs as a biomarker for Parkinson’s disease. Park Relat Disord.

[75] Béres NJ, Kiss Z, Sztupinszki Z, Lendvai G, Arató A, Sziksz E (2017). Altered mucosal expression of microRNAs in pediatric patients with inflammatory bowel disease. Dig Liver Dis.

[76] Wu F, Zikusoka M, Trindade A, Dassopoulos T, Harris ML, Bayless TM (2008). MicroRNAs are differentially expressed in ulcerative colitis and alter expression of macrophage inflammatory peptide-2α. Gastroenterology.

[77] Chuang AY, Chuang JC, Zhai Z, Wu F, Kwon JH (2014). NOD2 expression is regulated by microRNAs in colonic epithelial HCT116 cells. Inflamm Bowel Dis.

[78] Maeda S, Ohno K, Nakamura K, Uchida K, Nakashima K, Fukushima K (2011). Quantification of chemokine and chemokine receptor gene expression in duodenal mucosa of dogs with inflammatory bowel disease. Vet Immunol Immunopathol.

[79] Montenegro D, Romero R, Pineles BL, Tarca AL, Kim YM, Draghici S (2007). Differential expression of microRNAs with progression of gestation and inflammation in the human chorioamniotic membranes. Am J Obstet Gynecol.

[80] Ohlsson Teague EMC, Van der Hoek KH, Van der Hoek MB, Perry N, Wagaarachchi P, Robertson SA (2009). MicroRNA-regulated pathways associated with endometriosis. Mol Endocrinol.

[81] Fulci V, Scappucci G, Sebastiani GD, Giannitti C, Franceschini D, Meloni F (2010). miR-223 is overexpressed in T-lymphocytes of patients affected by rheumatoid arthritis. Hum Immunol.

[82] Schönauen K, Le N, von Arnim U, Schulz C, Malfertheiner P, Link A (2018). Circulating and fecal microRNAs as biomarkers for inflammatory bowel diseases. Inflamm Bowel Dis.

[83] Wang H, Zhang S, Yu Q, Yang G, Guo J, Li M (2016). Circulating microRNA223 is a new biomarker for inflammatory bowel disease. Medicine (Baltimore).

[84] Johnnidis JB, Harris MH, Wheeler RT, Stehling-Sun S, Lam MH, Kirak O (2008). Regulation of progenitor cell proliferation and granulocyte function by microRNA-223. Nature.

[85] Duttagupta R, DiRienzo S, Jiang R, Bowers J, Gollub J, Kao J (2012). Genome-wide maps of circulating miRNA biomarkers for ulcerative colitis. PLoS One.

[86] Allenspach K, Wieland B, Gröne A, Gaschen F (2007). Chronic enteropathies in dogs: evaluation of risk factors for negative outcome. J Vet Intern Med.

[87] García-Sancho M, Rodríguez-Franco F, Sainz A, Mancho C, Rodríguez A (2007). Evaluation of clinical, macroscopic, and histopathologic response to treatment in nonhypoproteinemic dogs with lymphocytic-plasmacytic enteritis. J Vet Intern Med.

[88] Slovak JE, Wang C, Sun Y, Otoni C, Morrison J, Deitz K (2015). Development and validation of an endoscopic activity score for canine inflammatory bowel disease. Vet J.

[89] Washabau RJ, Day MJ, Willard MD, Hall EJ, Jergens AE, Mansell J (2010). Endoscopic, biopsy, and histopathologic guidelines for the evaluation of gastrointestinal inflammation in companion animals. J Vet Intern Med.

