# Supplementary material for: Colonic mucosal and serum expression of microRNAs in canine large intestinal inflammatory bowel disease
Source: BMC Vet Res. 2020 Feb 22;16:69. doi: 10.1186/s12917-020-02287-6 (PMC7035774; doi:10.1186/s12917-020-02287-6)
Supplement: Supplementary file 1 — Additional file 1: Table S1. Correlations of the relative expression of miR-16, miR-21, miR-122, miR-146a, miR-147, miR-185, miR-192 and miR-223 in the serum (n = 21) and the colonic mucosa (n = 26) with inflammatory and morphologic histopathologic features score according to the World Small Animal Veterinary Association (WSAVA) GI Standardization Group guidelines in defining inflammation involving the colon of dogs with large intestinal inflammatory bowel disease (IBD). LP = lamina propria, miR = microRNA. [file 12917_2020_2287_MOESM1_ESM.docx]

|  | | Surface epithelial injury | Crypt hyperplasia | Crypt dilation and distortion | Mucosal fibrosis and atrophy | LP lymphocytes/ plasma cells | LP eosinophils | LP neutrophils | LP macrophages |
| --- | --- | --- | --- | --- | --- | --- | --- | --- | --- |
| Serum | miR-16 | *r*(21) = 0.016  *p* = 0.946 | *r*(21) = 0.120  *p* = 0.604 | *r*(21) = -0.096  *p* = 0.678 | *r*(21) = 0.427  *p* = 0.053 | *r*(21) = -0.147  *p* = 0.524 | *r*(21) = -0.308  *p* = 0.174 | *r*(21) = -0.154  *p* = 0.505 | *r*(21) = -0.008  *p* = 0.972 |
|  | miR-21 | *r*(21) = -0.252  *p* = 0.270 | *r*(21) = 0.090  *p* = 0.698 | *r*(21) = 0.145  *p* = 0.531 | *r*(21) = -0.039  *p* = 0.173 | *r*(21) = -0.297  *p* = 0.191 | *r*(21) = -0.482  *p* = 0.027 | *r*(21) = -0.195  *p* = 0.399 | *r*(21) = -0.081  *p* = 0.727 |
|  | miR-122 | *r*(21) = -0.239  *p* = 0.297 | *r*(21) = -0.322  *p* = 0.155 | *r*(21) = -0.228  *p* = 0.319 | *r*(21) = -0.140  *p* = 0.544 | *r*(21) = -0.006  *p* = 0.980 | *r*(21) = -0.241  *p* = 0.292 | *r*(21) = 0.194  *p* = 0.399 | *r*(21) = 0.178  *p* = 0.440 |
|  | miR-146a | *r*(21) = 0.005  *p* = 0.985 | *r*(21) = 0.249  *p* = 0.276 | *r*(21) = -0.096  *p* = 0.678 | *r*(21) = -0.099  *p* = 0.668 | *r*(21) = -0.069  *p* = 0.766 | *r*(21) = -0.281  *p* = 0.216 | *r*(21) = 0.122  *p* = 0.600 | *r*(21) = 0.097  *p* = 0.675 |
|  | miR-147 | *r*(21) = -0.056  *p* = 0.811 | *r*(21) = 0.081  *p* = 0.727 | *r*(21) = -0.039  *p* = 0.866 | *r*(21) = 0.026  *p* = 0.910 | *r*(21) = -0.147  *p* = 0.525 | *r*(21) = 0.241  *p* = 0.292 | *r*(21) = -0.405  *p* = 0.069 | *r*(21) = 0.113  *p* = 0.625 |
|  | miR-185 | *r*(21) = 0.082  *p* = 0.724 | *r*(21) = 0.105  *p* = 0.650 | *r*(21) = 0.264  *p* = 0.248 | *r*(21) = 0.004  *p* = 0.987 | *r*(21) = 0.065  *p* = 0.780 | *r*(21) = -0.121  *p* = 0.602 | *r*(21) = 0.000  *p* = 1.000 | *r*(21) = 0.008  *p* = 0.972 |
|  | miR-192 | *r*(21) = -0.261  *p* = 0.252 | *r*(21) = 0.005  *p* = 0.985 | *r*(21) = 0.152  *p* = 0.510 | *r*(21) = -0.318  *p* = 0.161 | *r*(21) = -0.169  *p* = 0.463 | *r*(21) = -0.188  *p* = 0.415 | *r*(21) = -0.170  *p* = 0.461 | *r*(21) = -0.251  *p* = 0.272 |
|  | miR-223 | *r*(21) = 0.362  *p* = 0.107 | *r*(21) = 0.307  *p* = 0.176 | *r*(21) = 0.141  *p* = 0.543 | *r*(21) = 0.093  *p* = 0.689 | *r*(21) = -0.050  *p* = 0.829 | *r*(21) = -0.188  *p* = 0.415 | *r*(21) = -0.3  *p* = 0.187 | *r*(21) = -0.429  *p* = 0.052 |
| Colonic mucosa | miR-16 | *r*(26) = 0.163  *p* = 0.480 | *r*(26) = -0.136  *p* = 0.558 | *r*(26) = -0.026  *p* = 0.912 | *r*(26) = 0.231  *p* = 0.314 | *r*(26) = 0.231  *p* = 0.313 | *r*(26) = -0.121  *p* = 0.602 | *r*(26) = -0.032  *p* = 0.889 | *r*(26) = -0.235  *p* = 0.305 |
|  | miR-21 | *r*(26) = -0.117  *p* = 0.613 | *r*(26) = 0.301  *p* = 0.184 | *r*(26) = 0.156  *p* = 0.500 | *r*(26) = -0.186  *p* = 0.420 | *r*(26) = 0.113  *p* = 0.625 | *r*(26) = -0.228  *p* = 0.321 | *r*(26) = -0.162  *p* = 0.483 | *r*(26) = -0.340  *p* = 0.131 |
|  | miR-122 | *r*(26) = 0.789  *p* <0.0005 | *r*(26) = 0.293  *p* = 0.197 | *r*(26) = 0.339  *p* = 0.133 | *r*(26) = 0.214  *p* = 0.352 | *r*(26) = 0.241  *p* = 0.292 | *r*(26) = -0.335  *p* = 0.138 | *r*(26) = 0.121  *p* = 0.600 | *r*(26) = 0.170  *p* = 0.461 |
|  | miR-146a | *r*(26) = 0.152  *p* = 0.512 | *r*(26) = 0.372  *p* = 0.097 | *r*(26) = -0.079  *p* = 0.732 | *r*(26) = -0.005  *p* = 0.982 | *r*(26) = 0.191  *p* = 0.406 | *r*(26) = -0.040  *p* = 0.863 | *r*(26) = -0.478  *p* = 0.028 | *r*(26) = -0.235  *p* = 0.305 |
|  | miR-147 | *r*(26) = -0.036  *p* = 0.877 | *r*(26) = -0.232  *p* = 0.311 | *r*(26) = -0.121  *p* = 0.602 | *r*(26) = 0.147  *p* = 0.524 | *r*(26) = -0.168  *p* = 0.467 | *r*(26) = -0.107  *p* = 0.644 | *r*(26) = -0.202  *p* = 0.379 | *r*(26) = 0.073  *p* = 0.754 |
|  | miR-185 | *r*(26) = 0.035  *p* = 0.882 | *r*(26) = -0.099  *p* = 0.670 | *r*(26) = 0.149  *p* = 0.520 | *r*(26) = -0.436  *p* = 0.048 | *r*(26) = -0.146  *p* = 0.529 | *r*(26) = -0.161  *p* = 0.486 | *r*(26) = -0.081  *p* = 0.727 | *r*(26) = -0.437  *p* = 0.057 |
|  | miR-192 | *r*(26) = -0.201  *p* = 0.381 | *r*(26) = -0.276  *p* = 0.226 | *r*(26) = 0.019  *p* = 0.936 | *r*(26) = -0.375  *p* = 0.094 | *r*(26) = -0.294  *p* = 0.195 | *r*(26) = 0.214  *p* = 0.351 | *r*(26) = -0.251  *p* = 0.272 | *r*(26) = -0.300  *p* = 0.185 |
|  | miR-223 | *r*(26) = -0.039  *p* = 0.866 | *r*(26) = -0.275  *p* = 0.227 | *r*(26) = -0.292  *p* = 0.198 | *r*(26) = 0.024  *p* = 0.919 | *r*(26) = -0.192  *p* = 0.406 | *r*(26) = 0.161  *p* = 0.486 | *r*(26) = 0.081  *p* = 0.727 | *r*(26) = 0.186  *p* = 0.418 |
