# Supplementary material for: Colonic mucosal and serum expression of microRNAs in canine large intestinal inflammatory bowel disease
Source: BMC Vet Res. 2020 Feb 22;16:69. doi: 10.1186/s12917-020-02287-6 (PMC7035774; doi:10.1186/s12917-020-02287-6)
Supplement: Supplementary file 2 — Additional file 2: Table S2. Correlations of the relative expression of miR-16, miR-21, miR-122, miR-146a, miR-147, miR-185, miR-192 and miR-223 in the serum (n = 21) and the colonic mucosa (n = 26) with CCECAI and colonoscopy score of dogs with large intestinal inflammatory bowel disease (IBD). CCECAI = canine chronic enteropathy clinical activity index, miR = microRNA. [file 12917_2020_2287_MOESM2_ESM.docx]

|  | | CCECAI score | Colonoscopy score | Colonic mucosa |
| --- | --- | --- | --- | --- |
| Serum | miR-16 | *r*(21) = -0.048  *p* = 0.836 | *r*(21) = -0.016  *p* = 0.946 | *r*(21) = 0.361  *p* = 0.108 |
|  | miR-21 | *r*(21) = -0.248  *p* = 0.279 | *r*(21) = -0.022  *p* = 0.925 | *r*(21) = 0.101  *p* = 0.664 |
|  | miR-122 | *r*(21) = 0.030  *p* = 0.897 | *r*(21) = 0.274  *p* = 0.230 | *r*(21) = -0.398  *p* = 0.074 |
|  | miR-146a | *r*(21) = -0.024  *p* = 0.919 | *r*(21) = 0.094  *p* = 0.686 | *r*(21) = -0.104  *p* = 0.047 |
|  | miR-147 | *r*(21) = 0.025  *p* = 0.914 | *r*(21) = 0.313  *p* = 0.168 | *r*(21) = 0.186  *p* = 0.420 |
|  | miR-185 | *r*(21) = -0.053  *p* = 0.821 | *r*(21) = -0.385  *p* = 0.085 | *r*(21) = 0.305  *p* = 0.179 |
|  | miR-192 | *r*(21) = -0.466  *p* = 0.033 | *r*(21) = 0.079  *p* = 0.735 | *r*(21) = 0.033  *p* = 0.887 |
|  | miR-223 | *r*(21) = -0.184  *p* = 0.425 | *r*(21) = -0.491  *p* = 0.024 | *r*(21) = -0.115  *p* = 0.618 |
| Colonic mucosa | miR-16 | *r*(26) = -0.119  *p* = 0.607 | *r*(26) = -0.026  *p* = 0.913 |  |
|  | miR-21 | *r*(26) = -0.172  *p* = 0.456 | *r*(26) = 0.193  *p* = 0.402 |  |
|  | miR-122 | *r*(26) = -0.129  *p* = 0.578 | *r*(26) = -0.075  *p* = 0.745 |  |
|  | miR-146a | *r*(26) = -0.291  *p* = 0.200 | *r*(26) = -0.408  *p* = 0.066 |  |
|  | miR-147 | *r*(26) = -0.237  *p* = 0.301 | *r*(26) = 0.085  *p* = 0.715 |  |
|  | miR-185 | *r*(26) = -0.559  *p* = 0.008 | *r*(26) = -0.426  *p* = 0.054 |  |
|  | miR-192 | *r*(26) = -0.301  *p* = 0.185 | *r*(26) = -0.134  *p* = 0.562 |  |
|  | miR-223 | *r*(26) = 0.285  *p* = 0.211 | *r*(26) = -0.111  *p* = 0.632 |  |
